# Vitamin D status predicts reproductive fitness in a wild sheep population

**DOI:** 10.1038/srep18986

**Published:** 2016-01-13

**Authors:** Ian Handel, Kathryn A. Watt, Jill G. Pilkington, Josephine M. Pemberton, Alastair Macrae, Philip Scott, Tom N. McNeilly, Jacqueline L. Berry, Dylan N. Clements, Daniel H. Nussey, Richard J. Mellanby

**Affiliations:** 1 Royal (Dick) School of Veterinary Studies and The Roslin Institute, Division of Veterinary Clinical Studies, The University of Edinburgh, Hospital for Small Animals, Easter Bush Veterinary Centre, Roslin, Midlothian, EH25 9RG UK; 2 Institutes of Evolutionary Biology School of Biological Sciences, University of Edinburgh, Edinburgh, UK; 3 Infection and Immunity Research, School of Biological Sciences, University of Edinburgh, Edinburgh, UK; 4 Moredun Research Institute, Midlothian, UK; 5 Specialist Assay Laboratory, 2nd Floor, CSB3, Manchester, UK

## Abstract

Vitamin D deficiency has been associated with the development of many human diseases, and with poor reproductive performance in laboratory rodents. We currently have no idea how natural selection directly acts on variation in vitamin D metabolism due to a total lack of studies in wild animals. Here, we measured serum 25 hydroxyvitamin D (25(OH)D) concentrations in female Soay sheep that were part of a long-term field study on St Kilda. We found that total 25(OH)D was strongly influenced by age, and that light coloured sheep had higher 25(OH)D_3_ (but not 25(OH)D_2_) concentrations than dark sheep. The coat colour polymorphism in Soay sheep is controlled by a single locus, suggesting vitamin D status is heritable in this population. We also observed a very strong relationship between total 25(OH)D concentrations in summer and a ewe’s fecundity the following spring. This resulted in a positive association between total 25(OH)D and the number of lambs produced that survived their first year of life, an important component of female reproductive fitness. Our study provides the first insight into naturally-occurring variation in vitamin D metabolites, and offers the first evidence that vitamin D status is both heritable and under natural selection in the wild.

Traditionally, the main physiological role of vitamin D has been considered to be in the development and maintenance of skeletal health. Vitamin D deficiency was demonstrated to be the cause of rickets nearly a century ago but over the past two decades, numerous studies have linked vitamin D deficiency to the development of a wide range of non-skeletal disorders, including the development of numerous inflammatory, neoplastic and autoimmune diseases^1^,^2^,^3^,^4^. Considerable variation has typically been observed in circulating levels of vitamin D metabolites within healthy human populations^5^,^6^,^7^. This variation has been linked to environmental and phenotypic influences including diet, exposure to sunlight and skin colour, and has a heritable component^8^,^9^,^10^. At least part of the genetic variation observed in vitamin D status is likely to have its origin in our evolutionary past^11^. However, there remains considerable debate over the importance of potential fitness consequences of vitamin D deficiency in human evolution, in particular with respect to evolution of skin colour^11^. Epidemiological studies have linked vitamin D deficiency with all-cause mortality in humans^12^,^13^,^14^, while laboratory experiments using rodents have linked deficiency to infertility, reduced pregnancy rates and litter sizes^15^,^16^,^17^,^18^. This suggests vitamin D metabolism may influence two central components of Darwinian fitness: survival and reproductive success. However, we currently have no idea how natural selection really acts on variation in vitamin D metabolism due to a total lack of studies in the wild. Here, we present the first evidence linking variation in vitamin D metabolites and reproductive fitness from a natural population.

Vitamin D can be obtained from dietary sources or from the endogenous synthesis of vitamin D_3_ which occurs after photolytic conversion of 7-dehydrocholesterol, located in the dermal fibroblasts and epidermal keratinocytes, by the ultraviolet B component of sunlight^19^. Nutritional forms of vitamin D consist of plant derived vitamin D_2_ or vitamin D_3_ which is found in oily fish, eggs and liver. Vitamin D undergoes hydroxylation in the liver to 25 hydroxyvitamin D (25(OH)D) and is then converted into the active metabolite, 1,25 dihydroxyvitamin D, (1,25(OH) _2_D) in the kidneys by renal 1-α hydroxylase (CYP27B1)^19^. Due to the longer half-life and higher serum concentrations of 25(OH)D compared to 1,25(OH)_2_D, serum concentrations of 25(OH)D are the most widely used assessment of vitamin D status^19^. In the present study, we measured concentrations of 25(OH)D_2_ and 25(OH)D_3_ in serum collected from a group of female Soay sheep (*Ovis aries*) which were part of the long-term study in the Village Bay area on the island of Hirta in the St Kilda archipelago. Our aims were: (i) to test whether and how age and other available phenotypic traits, including coat colour, immunological measures, parasite burden and body mass, were associated with 25(OH)D_2_ and 25(OH)D_3_; (ii) to determine whether vitamin D concentrations predicted important reproductive fitness traits, including fecundity, offspring birth weight and offspring first year survival.

Soay sheep are descendants of domestic sheep that were present throughout the northwest of Europe during the Bronze age, and probably reached the St Kilda archipelago 3000–4000 years ago^20^. Previous studies have reported that 25(OH)D concentrations can be influenced by skin pigmentation in humans^21^,^22^, and so it is salient that Soay sheep can have either a dark or light coat colour phenotype (Fig. 1).

This polymorphism is known to be under the control of a single genetic locus (TYRP1), and occurs on St Kilda at an approximate 3 dark: 1 light ratio^23^. Soay sheep have been resident to the St Kilda archipelago for thousands of years, but were resident only on the smaller island of Soay until 1930 when around one hundred were moved to the (then uninhabited) larger island of Hirta which is where Village Bay and our study area is located. This population has been the subject of long term individual-based monitoring since 1985, and have been completely unmanaged and unpredated since the 1930s^20^. Sheep resident to Village Bay are captured and marked at birth and subsequently closely monitored and regularly captured, providing exceptionally detailed reproductive life history and survival data. They are also subject to variable and often very strong natural selection, characterized in particular by irregular ‘crash’ winters when upwards of 60% of the population can perish due to interactions among food limitation, climate conditions and parasite infection (Fig. 2)^20^. Importantly, the relatively homogeneous (compared to humans) and unsupplemented vegetable diet of the sheep means circulating 25(OH)D_3_ will be of cutaneous origin, while 25(OH)D_2_ will be dietary in origin. Here, we test the phenotypic predictors of vitamin D status in wild Soay sheep and, in turn, its ability to predict annual reproductive fitness. We present the first documentation of an association between colouration and serum 25(OH)D_3_ concentrations from the wild, and the first evidence that higher levels of either 25(OH)D_3_ or 25(OH)D_3_ predict improved female fecundity and reproductive fitness under natural conditions.

## Results

Serum 25(OH)D_2_ and 25(OH)D_3_ concentrations were positively correlated (r = 0.58, t_(99)_ =7.12, p < 0.001; Fig. 3), although 25(OH)D_3_ concentration were on average 29.14 ng/ml (95% CI 25.98–32.29 ng/ml) higher and thus contributed considerably more to individual’s total 25(OH)D (Fig. 3). Age explained a considerable amount of variation in 25(OH)D concentrations (Fig. 4). Comparison of models with a smooth function of age (implemented using a generalised additive model) and a three-level categorisation of age (lambs, adults aged 1–6 years, and geriatrics aged 7 years or more) revealed that the latter adequately described variation in all three vitamin D measures (ΔAIC was lower for all categorical models compared to the respective general additive models: 25(OH)D_2_ =−5.86, 25(OH)D_3_ = −7.96, total 25(OH)D = −8.31). The three category age function explained a considerable amount of variation in all three measures: r^2^ = 0.42, 0.31 and 0.39, respectively. For the 3 general additive models r^2^ was 25(OH)D_2_ = 0.28, 25(OH)D_3_ = 0.35, total 25(OH)D = 0.38.

Serum 25(OH)D_3_ concentrations were significantly higher in adults and geriatrics than lambs (p < 0.05 Tukey HSD test) and 25(OH)D_2_ concentration was significantly higher in adults than geriatrics and lambs (p < 0.05 Tukey HSD test). Serum 25(OH)D showed a decline from adult to geriatric age groups which was only statistically significant for 25(OH)D_2_ concentrations (mean difference 8.76 ng/ml, 95% CI 3.08–7.43, p < 0.001 unpaired T-Test).

Once age was accounted for, our main findings were that light coated ewes had higher 25(OH)D_3_ concentrations than dark ones (mean 25(OH)D_3_ concentration in light sheep was 8.11 ng/ml 95% CI 1.52–14.71 higher than in dark sheep, p = 0.014) (Fig. 5) and that 25(OH)D_2_ concentrations were positively associated with anti-*T. circumcincta* IgE levels (p = 0.003) and positively associated with weight (p = 0.046). T. circumcincta is an important gastrointestinal parasite of sheep and there is good evidence that on St Kilda these parasites exert a considerable selective pressure on Soay sheep^24^.

Final models of total 25(OH)D included a coat colour effect (P = 0.014, mean total 25(OH)D concentration 9.74 ng/ml 95% CI 1.78–17.70 higher in light than dark sheep), a positive anti-*T. circumcincta* IgE effect (p = 0.022), a negative anti-*T. circumcincta* IgG effect (p = 0.043), and an effect of age group (p < 0.001). All other terms were non-significant including faecal egg count (FEC) and were dropped from the model (Table 1).

Vitamin D status in August 2012 strongly predicted a female’s fecundity the following spring, but not lamb birth weight or lamb survival over the following 12 months (Fig. 6; Table 2). However, the fecundity effect alone meant that females with higher summer total vitamin D levels had a greater annual reproductive success (i.e. more lambs surviving to one year of age; Fig. 6; Table 2). Our final proportional odds regression model of female fecundity, prior to inclusion of Vitamin D measures, included only age group and IgETc (p = 0.008 & p = 0.009), as August weight and female coat colour did not improve model explanatory power once age was included (Table 2). Independent of age, 25(OH)D_2_, 25(OH)D_3_ or total 25(OH)D added separately to the model all improved model fit and positively predicted fecundity (p = 0.007, p < 0.001 & p < 0.001; Fig. 6, Table 2). Relaxing the assumption of proportional odds in each of these models did not result in a significant change in model fit, suggesting the assumption was supported (p = 0.394, p = 0.298 & p = 0.270 for 25(OH)D_2_, 25(OH)D_3_ and 25(OH)D total models respectively).

Our final model of lamb birth weight included mother’s age group, lamb sex, and lamb twin status, while capture age and mother’s coat colour did not improve model fit and were thus dropped (Table 2). None of the maternal vitamin D measures explained additional variation in lamb birth weight when added to this model (p = 0.670, p = 0.621 & p = 0.570 for 25(OH)D_2_, 25(OH)D_3_ and total 25(OH)D models respectively). The final model for lamb first winter survival included only lamb birth weight; mother’s age group and coat colour and lamb sex and twin status did not explain additional variation (Table 2). None of the maternal vitamin D measures explained additional variation in lamb first winter survival when added to this model (p = 0.403, p = 0.893 & p = 0.880 for 25(OH)D_2_, 25(OH)D_3_ and total 25(OH)D models respectively). Finally, none of the terms tested were significant predictors of female annual reproductive success (ARS) (Table S2), but we found that total 25(OH)D and 25(OH)D_3_ significantly and positively predicted ARS when fitted along in the model (p = 0.245, p = 0.033 & p = 0.045 for 25(OH)D_2_, 25(OH)D_3_ and total 25(OH)D total models respectively; Fig. 6, Table 2). The effect size of 25(OH)D_2_ on ARS was of a similar magnitude but was not statistically significant (log odds ratio 0.039, 0.026 & 0.020 for 25(OH)D_2_, 25(OH)D_3_ and total 25(OH)D total respectively; Fig. 6).

## Discussion

This study offers the first insight into the predictors of vitamin D status within an unmanaged animal population, and the first direct evidence of natural selection acting on circulating levels of vitamin D metabolites in the wild. We observed relationships between serum 25(OH)D_2_ and 25(OH)D_3_ concentrations and age, coat colour and anti-nematode IgE levels, which appear consistent with some previous findings from studies of humans and domestic animals^5^,^22^,^25^,^26^,^27^,^28^,^29^,^30^,^31^. Strikingly, we observed a very strong relationship between total 25(OH)D levels in summer and a ewe’s fecundity the following spring (Fig. 6). There was no evidence that vitamin D predicted lamb birth weight or over-winter survival, but the fecundity effect alone was sufficient to result in significantly improved annual reproductive success in females with higher vitamin D concentrations (Fig. 6). There is an evident reproductive fitness advantage to females with improved vitamin D status, which our findings suggest is mediated by an association with fecundity rather than post-parturition maternal care. This constitutes important support for the role of vitamin D in mammalian reproductive performance, and the first direct observation of natural selection acting on vitamin D metabolism in the wild.

In wild Soay sheep, cutaneously produced vitamin D_3_ contributed considerably more to total 25(OH)D concentrations than orally consumed vitamin D_2_ during the summer. Previous studies of vitamin D in humans and domestic animals are frequently confounded by regular vitamin D supplementation, as well as potentially dramatic variation in diet and exposure to sunlight^10^,^31^. The unsupplemented and relatively homogeneous vegetable diet of Soay sheep on St Kilda means that they did not have access to dietary sources of vitamin D_3_^32^. Thus, in our animals, all 25(OH)D_3_ must have been cutaneous in origin. It seems unlikely that variation in UV exposure can explain observed variation in 25(OH)D_3_ in Soay sheep, since there is a total lack of canopy cover on Hirta, and our samples were collected within a 2 week period in mid-summer. Our study therefore demonstrates that latitude alone is poor predictor of vitamin D_3_ status in wild animals; we have demonstrated that sheep of the same gender, coat colour and age living in a geographically constrained area can have wide fluctuations in serum 25(OH)D_3_ concentrations. Furthermore, we have demonstrated that vitamin D_3_ status is highly predictive of subsequent fecundity and annual reproductive success when latitude is fixed and opportunities to modify exposure to UV radiation are limited.

Serum 25(OH)D_3_ concentrations in Soay sheep were predicted by coat colour, independent of age, which adds to existing evidence for associations between pigmentation and vitamin D metabolism^21^,^22^, as well as providing strongly suggestive evidence that vitamin D_3_ variation is under genetic control under natural conditions. It was recently established that in Soay sheep, the light/dark coat colour polymorphism is the result of a single base pair substitution at a single genetic locus, TYRP1, at which the dark allele is dominant to light^23^. The locus itself is known to be under complex natural selection, with homozygous dark sheep at an apparent fitness disadvantage^33^. The increased 25(OH)D_3_ concentrations in light sheep is consistent with previously observed associations between light skin or wool pigmentation and vitamin D status in humans and domestic sheep^21^,^22^,^31^. However, it also implies that the recessive allele responsible for light colouration has either direct or indirect effects on vitamin D metabolism and thus, to some degree at least, that genetic factors underpin 25(OH)D_3_ variation in our study population. Human twin studies have demonstrated that vitamin D status has a heritable component^34^, and recent genome wide association studies of circulating 25(OH)D concentrations identified several genes which influenced vitamin D status in humans^8^,^9^. Our study provides the first evidence suggesting that genetic variation exists in vitamin D status in a wild animal.

Variation in serum 25(OH)D_2_ concentrations in the Soay ewes sampled here was also noteworthy and could reflect altered grazing behaviour between individuals as well as genetic factors. We found evidence of a positive correlation between 25(OH)D_2_ concentrations and plasma levels of IgE immunoglobulin against *T. circumcincta*. Development of a robust IgE response is important in preventing *T. circumcincta* parasites becoming established in the gut of domestic sheep^35^,^36^,^37^, and there is good evidence that on St Kilda these parasites exert a considerable selective pressure on Soay sheep^24^. Positive correlations between vitamin D status and antigen specific IgE responses have also been reported in human studies^25^,^26^, and vitamin D supplementation has also been shown to increase IgE production to whole worm antigen in patients with *Schistostoma haematobium* infection^30^.Thus, it is possible that ewes with raised vitamin D_2_ and IgE levels may show improved resistance to gastrointestinal nematode parasites. However, previous work suggests broadly positive associations between anti- *T. circumcincta* IgE levels and Strongyle FEC in adult Soay ewes, consistent with antibody levels reflecting parasite exposure rather than resistance^38^. It is therefore also possible that the association between IgE and vitamin D_2_ is driven by spatial heterogeneity in both vegetation and exposure to Strongyle larvae. Our sample size was insufficient to meaningfully test how variation in habitat within Village Bay might influence vitamin D status, but this is an important avenue for future study.

Summer vitamin D status predicted annual reproductive success over the following year in female Soay sheep. Importantly, we were able to demonstrate that this was due to associations between vitamin D concentrations and fecundity, rather than with lamb birth weight or lamb first year survival rates. Previous work on domestic animals and rodent models supports a link between vitamin D status and both fecundity and reproductive performance traits^16^,^17^,^39^,^40^,^41^. Although the mechanisms responsible for the association between vitamin D status and female fecundity documented in Soay sheep remains unknown, the strength of the relationship – which is considerably stronger than observed for other potential predictors of fecundity, such as age and weight – in what is a relatively small sample, suggests important links between vitamin D metabolism and reproductive fitness exist in natural populations of animals. However, our study provides only a first glimpse of the potentially complex way in which natural selection acts on vitamin D status in the wild. We have considered only a single year’s reproductive performance across the life times of individual ewes that can live well over a decade under natural conditions. Whether and how vitamin D status relates to longevity or lifetime reproductive fitness remains to be determined. Furthermore, our samples come from a year when sheep numbers were low and conditions were relatively benign on Hirta. Both vitamin D status itself and its relationship with fitness may change dramatically with environmental conditions in different years. Our work provides the first evidence that vitamin D status has a genetic basis and is under selection in the wild. Further long-term studies in the wild can help elucidate how natural selection shapes and maintains genetic variation in vitamin D status.

## Materials & Methods

### Field and laboratory data collection

**Figure 2:**
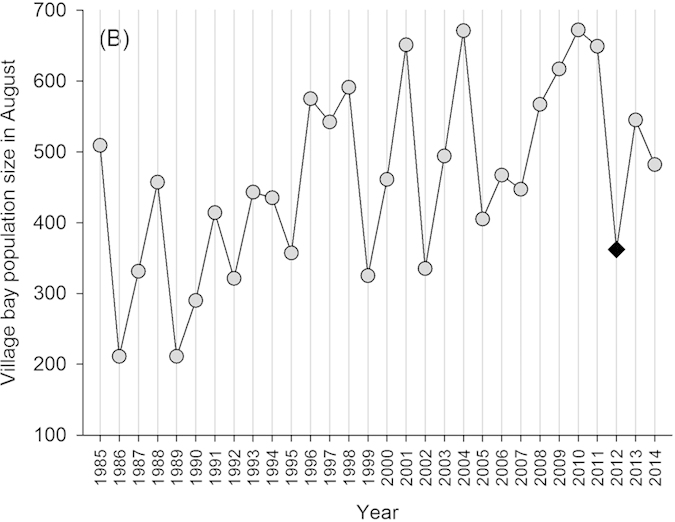
Annual population size of Soay sheep population in August in Village Bay area on the island of Hirta, with year of sample collection in this study highlighted as a black diamond.

Since 1985, the sheep resident in the Village Bay area on the island of Hirta have been the subject of long term individual-based monitoring. These animals are completely unmanaged and unpredated and their reproductive life histories and survival rates are closely tracked throughout the year. Ewes typically produce a single lamb each spring, although around 18% produce twins. Fecundity is strongly influenced by age: Soay ewes are mature in their first year but fecundity in lambs is low and environment-dependent, while most prime age adults (1–6 years) produce lambs each year and fecundity declines as individuals senesce from 7 years onwards. Lambs are caught, weighed and tagged within a few days of birth in spring, and are then monitored closely for the rest of their lives. Every August as many sheep as possible in the Village Bay study area are captured using temporary traps and weights and blood and faecal samples are taken. Natural mortality occurs in late winter as a result of poor nutrition, challenging climate conditions and infection with parasites, particularly gastro-intestinal nematodes^42^,^43^. The population dynamic is characterised by periods of low and rising sheep numbers followed by dramatic ‘crash winters’ when greater than 60% of the population can perish (Fig. 2). Regular censuses and searches of the study area through the late winter and early spring period mean that the majority of carcasses are found, and death dates can be reliably assigned to study animals^20^.

In this study we used data and samples collected from 101 ewes caught in August 2012. The survival and reproductive performance of these ewes was monitored over the following two years. August 2012 directly followed a population crash over the winter of 2011/2012 (Fig. 2), and since sheep numbers were relatively low and there was very low mortality in winter 2012/2013: only 4 of 101 ewes died. 77 of the surviving ewes were fecund and produced 93 lambs in spring 2013 (16 twin pairs, 61 singletons), of which 83 were caught and weighed at birth. Lamb neonatal mortality in 2013 was very low (83 of the 93 lambs survived to August) but lamb over-winter mortality was high (only 28 of the 93 survived their first winter) due to harsh winter weather in late 2013 and early 2014.

Individuals captured in August 2012 were weighed, faecal sampled, and 9ml of whole blood was collected into a heparinised tube and a further 5 ml into a serum tube and both were stored at 4 °C. Nematode eggs in faecal samples were counted shortly after collection, and strongyle faecal egg count (FEC) was estimated as the number of eggs per gram using a modified McMaster technique^44^. On St Kilda, five nematode species contribute to this count, the most abundant being *T. circumcincta*, *Trichostrongylus axei* and *Trichostrongylus vitrinus*, and FEC has previously been shown to correlate with weight and over-winter survival prospects in our study population^24^,^43^. Within 24 hours of collection, heparinised tubes were centrifuged at 3000 r.p.m. (approx. 1500g) for 10 min and the plasma layer removed and stored at −20 °C. These plasma samples were used in ELISA antibody assays to quantify concentrations of anti-*T. circumcinta* third larval stage (L3) IgG and IgE as previously described^38^. Anti-*T. circumcinta* antibody levels have previously been related to FEC, adult survival and subsequent fecundity in this population^38^,^45^.

Serum tubes were centrifuged at 3000 r.p.m. (approx. 1500g) for 10 min, and the serum was removed and stored at −20 °C. Serum concentrations of 25(OH)D_2_ and 25(OHD_3_ were determined by liquid chromatography tandem mass spectrophotometry (LC-MS/MS) using an ABSciex 5500 tandem mass spectrophotometer (Warrington, UK) and the Chromsystems (Munich, Germany) 25OHD kit for LC-MS/MS following the manufacturers’ instructions (intra- and inter-assay CV 3.7% and 4.8% respectively). This Supraregional Assay Service laboratory is accredited by CPA UK (CPA number 0865) and has been certified as proficient by the international Vitamin D Quality Assurance Scheme (DEQAS) (25). Total 25(OH)D is defined as the sum of 25(OH)D_2_ and 25(OH)D_3_. Serum calcium concentrations were measured on an automated wet chemistry analyser (KoneLab 60i; Thermo Clinical Labsystems, Vantaa, Finland). Only 7 out of 99 sheep with electrolyte results had a serum calcium concentration below 2mmol/l demonstrating that hypocalcaemia was rarely diagnosed in our cohort.

All experiments had local ethical approval and were performed in accordance with UK legislation. The experimental protocols were approved by the University of Edinburgh Animal Welfare and Ethics Review Body.

### Statistical analysis

**Figure 3:**
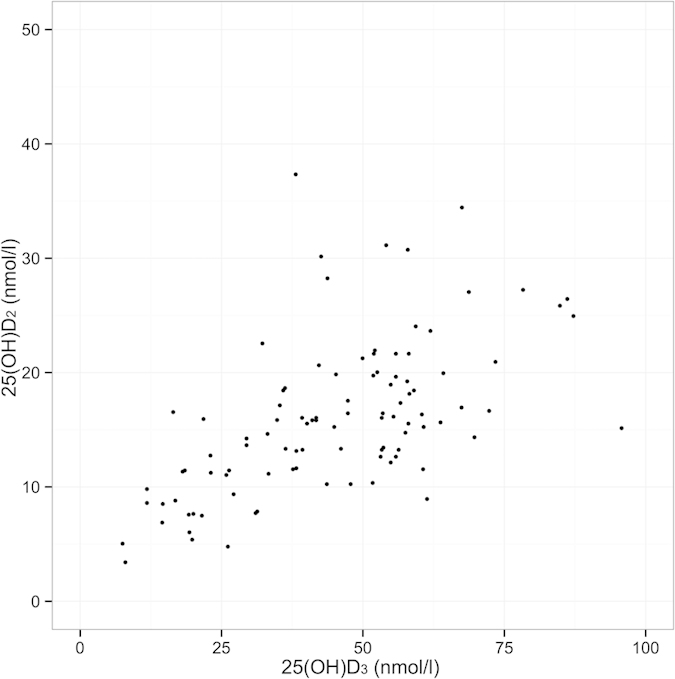
Scatterplot showing relationship between 25(OH)D_2_ and 25(OH)D_3_

**Figure 4:**
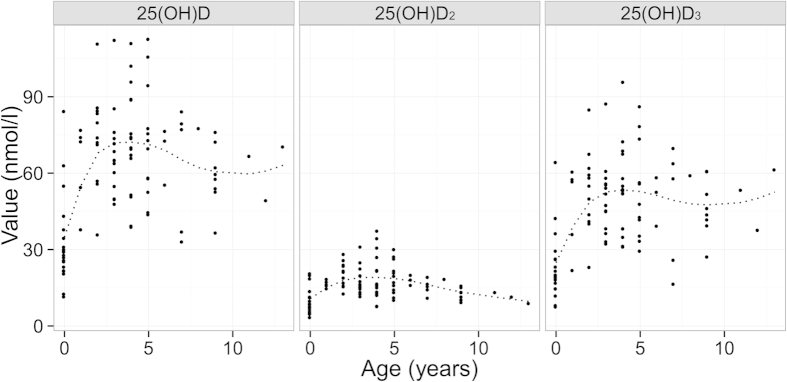
Association between age and serum concentrations of total 25(OH)D, 25(OH)D_2_ and 25(OH)D_3_. Individual results are shown as dots. Fitted general additive model shown as dotted line.

**Figure 5:**
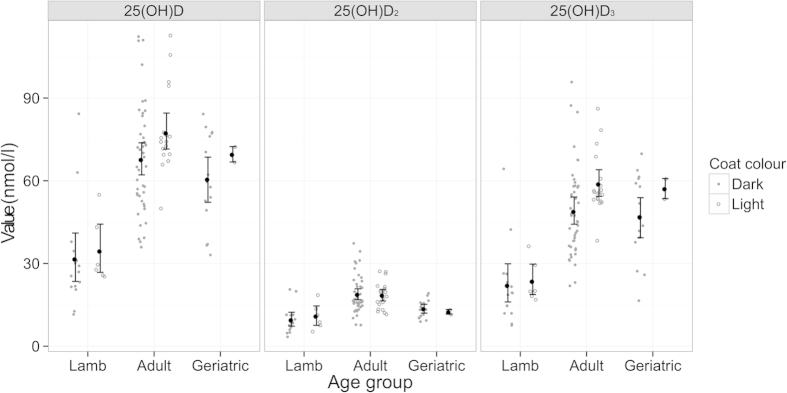
Association between 25(OH)D concentration, age and coat colour. Black points show mean with 95% confidence interval.

**Figure 6:**
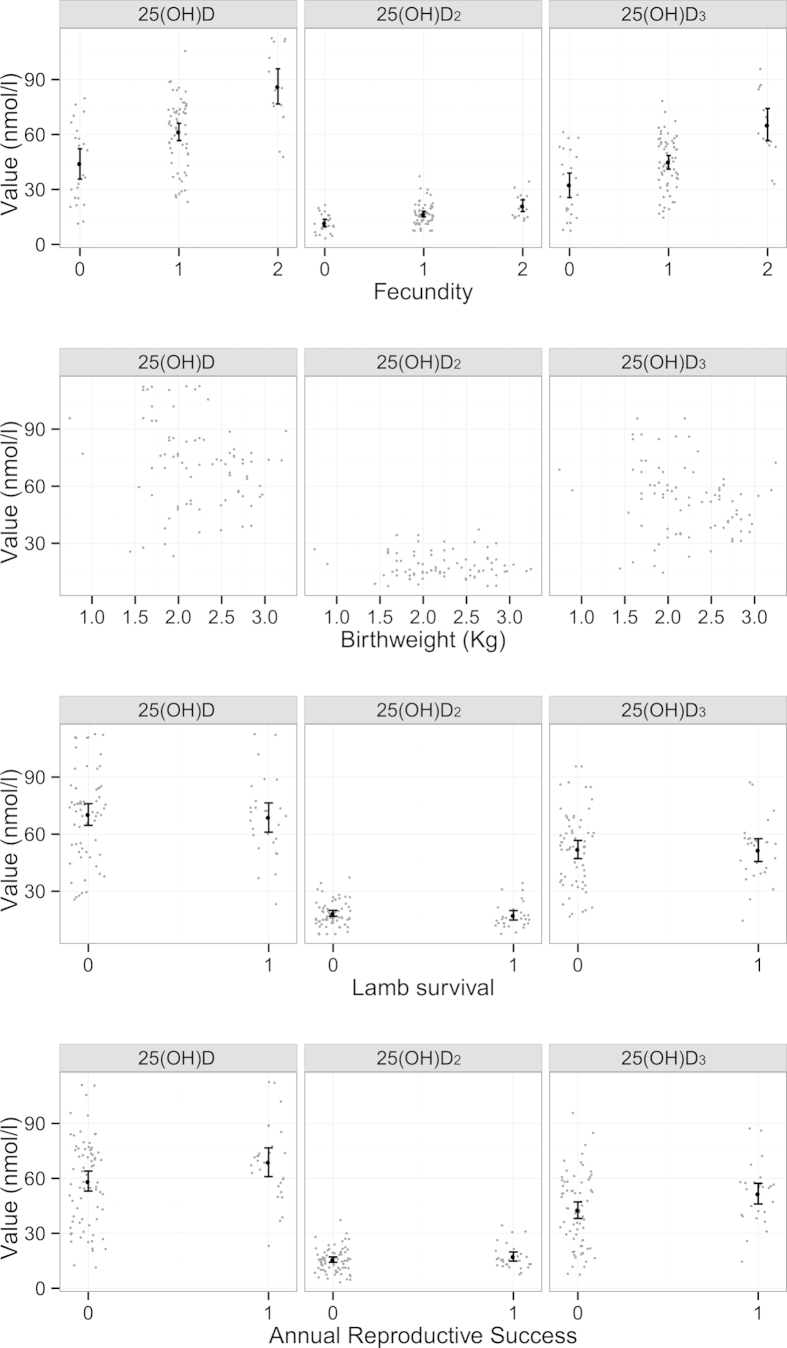
Association between fecundity (number of lambs born), Birthweight, Lamb survival to 4 months of age (conditional on birth) and Annual reproductive success and serum concentrations of total 25(OH)D, 25(OH)D_2_ and 25(OH)D_3_. Points are randomly ‘jittered’ on the horizontal axis, to separate points, for all variables except for birth weight.

We began by examining the distributions of our three vitamin D measures, and testing the difference in mean concentrations of 25(OH)D_2_ and 25(OH)D_3_ within samples, as well as their correlation (paired t-test, Pearson’s product moment correlation). We found a strong positive correlation between 25(OH)D_2_ and 25(OH)D_3_ concentrations (Fig. 3). In our analysis we examined total 25(OH)D and its two constituent measures separately in all further models. We first tested whether and how available phenotypic data predicted 25(OH)D status using separate linear models of total 25(OH)D, 25(OH)D_2_ and 25(OH)D_3_. Since dramatic age-related variation in demographic rates, biometrics, immune and parasitological measures have been previously observed in Soay sheep, which characteristically show increases from lamb to adult (1–6 years) followed by senescent declines from 7 years onwards^42^,^46^,^47^, we began by examining age effects on vitamin D status. We fitted a smoother for age, implemented using a generalised additive model, and compared the fit of this unconstrained age function to models using our *a priori* expectation of a three-level age function comprising lamb, adult (1–6 years) and geriatrics (7 years or more) groups using AIC.

Having established that, as expected, age explained a considerable proportion of variation in all three vitamin D measures, and that our three-level age function adequately captured age-related variation (see Results), we proceeded to test additional effects in these models whilst retaining age group in all models. Since we did not have complete observations for FEC (88 observations) we tested this effect separately, comparing models of each vitamin D measure with accordingly restricted sample sizes which either excluded or included FEC, using likelihood ratio tests (LRTs). Since FEC did not significantly improved model fit, we proceeded, using a full data set, by adding August weight and anti-*T. circumcincta* IgG and IgE levels to models of total 25(OH)D, 25(OH)D_2_ and 25(OH)D_3_. We simplified models by dropping each term in the model and removing the one producing the lowest LRT statistic, until only terms that were significant at the p < 0.05 level remained. We then checked for erroneous term deletions by separately adding each term back into the final model, and confirming this did not improve model fit using LRTs.

The relationship between candidate predictors and subsequent fecundity was investigated by treating ewe fecundity as an ordinal outcome (0, 1, or 2 lambs born the following spring). Previous studies have shown that ewe fecundity is strongly predicted by ewe age and, to a lesser degree, by her weight the previous August^20^. We used proportional odds logistic regression (POLR) to model the relationship between fecundity and candidate predictors. POLR models the ratio of the odds that an individual has a particular outcome compared to the next ‘lower’ outcome as being dependent on a linear combination of predicting variables. Here, the proportional odds assumption assumes that a change in covariates has the same impact on the odds ratio of producing two lambs vs. one lamb as it does on the odds ratio of producing one lamb vs. no lambs. This assumption simplifies the fitted model and if valid increases the precision of estimated relationships. The ordinal regression model’s assumptions can be relaxed, at the expense of inclusion of extra parameters, by allowing the influence of covariates to be different between no lambs and one lamb compared with one and two lambs. We tested the assumption of proportional odds by comparison with non-proportional odds models using a likelihood ratio test. In our initial POLR model we included age group, weight, coat colour, anti-*T. circumcincta* IgE and IgG. We excluded 25(OH)D from the initial model. This model was simplified using likelihood ratio tests as described earlier in the methods. We then added each individual vitamin D component into the final model to assess whether it added predictive value to the model using likelihood ratio tests.

Fecundity is only one aspect of a ewe’s annual reproductive success or fitness. Lamb birth weight has important fitness consequences, as it strongly predicts lamb survival which, in turn, has important consequences for both offspring and maternal reproductive fitness^48^. We therefore tested whether and how vitamin D status predicts lamb birth weight and lamb first winter survival. Birth weights were normally distributed and addressed using standard linear models. Previous work has demonstrated effects of capture age (in days), offspring sex, twin status and mother’s age on birth weight^48^. Therefore, we initially tested the effects of these terms, as well as mother’s coat colour and anti-*T. circumcincta* IgE levels (since they predicted 25(OH)D_3_ and 25(OH)D_2_ concentrations, respectively), on offspring birth weight, using the model simplification approach described above. Having determined a final model for birth weight, we separately added 25(OH)D_2_, 25(OH)D_3_ and total 25(OH)D to the model to test their importance. Lamb neonatal survival rates were so high in our sample (89%) that they were not deemed worthy of further analysis, but first winter survival rates were relatively low (30%) and we therefore modelled winter survival as a binary variable (1 = survived, 0 = died) using generalised linear models with a binomial error distribution. We tested the same initial terms as in the birth weight models, except we substituted birth weight itself for capture age, and proceeded to test vitamin D measures against the final model as described for birth weight. Finally, we tested the overall effects of vitamin D on ewe annual reproductive success (ARS) – which we defined as the number of lambs produced by a female sheep which survived their first year of life. Since female ARS in our sample was either zero or one, we coded this as a binary variable and fitted a generalised linear model fitted with a binomial error distribution, but otherwise modelled this trait as we had done for fecundity. All models were fitted using the R statistical system.

## Additional Information

**How to cite this article**: Handel, I. *et al.* Vitamin D status predicts reproductive fitness in a wild sheep population. *Sci. Rep.* **6**, 18986; doi: 10.1038/srep18986 (2016).

## Figures and Tables

**Figure 1:**
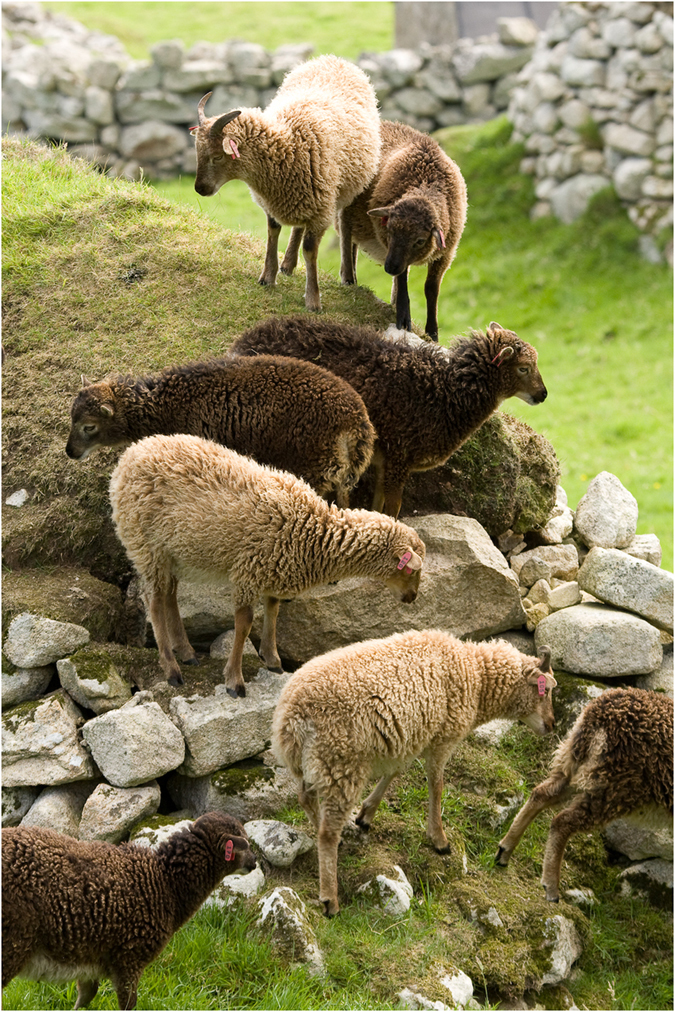
Dark and light coated Soay sheep in the Village Bay area of St Kilda (photo credit Arpat Ozgul).

**Table 1:** Results of regression models of 25(OH)D components including likelihood ratio test (LRT) p-values, estimates and standard error of estimate in brackets.

		Total 25(OH)D	25(OH)D_2_	25(OH)D_3_
Final model	Coat colour (light vs dark)	P = 0.014 9.744 (4.008)	–	P = 0.014 8.112 (3.323)
	IgE	P = 0.022 38.848 (12.764)	P = 0.003 8.755 (2.964)	–
	IgG	P = 0.043 −18.679 (9.460)	–	
	Weight	–	P = 0.046 0.451 (0.229)	–
	Age Group (adult vs lamb) (geriatric vs lamb)	p < 0.001 36.706 (4.787) 25.937 (6.263)	p < 0.001 3.093 (2.592) -4.563 (3.518)	p < 0.001 29.250 (3.681) 27.002 (4.799)
Dropped terms	Coat colour	–	P = 0.680	–
	IgE	–	—	P = 0.080
	IgG	–	P = 0.227	P = 0.792
	Weight	P = 0.870	–	P = 0.545
Addition of FEC to model using only Age Group		P = 0.123	P = 0.224	P = 0.161

LRT of addition of previous dropped terms and addition of faecal egg count to a model using only age group are shown in the lower sections.

**Table 2:** Results from regression models of fecundity, annual reproductive success, lamb birthweight and lamb over winter survival showing LRT p-values, estimates and (standard errors).

		Fecundity	Annual reproductive success	Birthweight	Lamb survival
Final model	Age group	p = 0.008 Adult: 1.41 (0.56) Geri: 0.16 (0.72)		P < 0.001 Adult: 0.755 (0.152)	
	IgETc	p = 0.009 3.03 (1.19)			
	Lamb sex			p = 0.016 Female: 0.187 (0.078)	
	Twin			P < 0.001 Twin: −0.661 (0.080)	
	Birthweight				p = 0.002 1.606 (0.571)
Dropped terms	Age group		p = 0.073		p = 0.936
	Weight	p = 0.122	p = 0.091		
	Coat colour	p = 0.287	p = 0.797		p = 0.875
	IgETc	-	p = 0.368		
	IgGTc	p = 0.292	p = 0.345		
	Lamb sex				p = 0.423
	Twin				p = 0.489
Addition of 25(OH)D	25(OH)D_2_	p = 0.007 0.106 (0.040)	p = 0.245	p = 0.670	p = 0.403
	25(OH)D_3_	P < 0.001 0.066 (0.017)	p = 0.033 0.026 (0.013)	p = 0.621	p = 0.893
	Total 25(OH)D	P < 0.001 0.058 (0.014)	p = 0.045 0.020 (0.010)	p = 0.570	p = 0.880

Lower sections show dropped terms from models (LRT p-values) and effect of adding 25(OH)D components to models (LRT p-values). Fecundity, lamb survival and annual reproductive success estimates are log odds ratios. Birth weight estimates are linear model coefficients.
